# Application of Additive Manufacturing in Assisted Reproductive Techniques: What Is the Evidence? A Clinical and Technical Systematic Review of the Literature

**DOI:** 10.3390/medicina60111889

**Published:** 2024-11-18

**Authors:** Adamantia Kontogeorgi, Ioannis Boutas, Gkalia Tsangkalova, Pantelis Messaropoulos, Nektarios I. Koufopoulos, Roxana Schwab, Antonis Makrigiannakis, Magda Zanelli, Andrea Palicelli, Maurizio Zizzo, Giuseppe Broggi, Rosario Caltabiano, Sophia N. Kalantaridou

**Affiliations:** 1 Medical School, University of Crete, 13 Andrea Kalokairinoy Ave., 715 00 Giofirakia, Greece; ad.kontogewrgi@gmail.com (A.K.); makrigia@med.uoc.gr (A.M.); 2 Breast Unit, Rea Maternity Hospital, 383 Andrea Siggrou Ave., 175 64 Paleo Faliro, Greece; drboutas@gmail.com; 3 Serum IVF Fertility Center, 26, Leof. Sofokli Venizelou Av., 141 23 Likovrisi, Greece; gkalia.tsangkalova@ivfserum.com; 4 Gynecology Clinic, Mediteraneo Hospital, Hlias 8-12, 166 75 Glyfada, Greece; pantelismess@gmail.com; 5 Second Pathology Department, Medical School, National and Kapodistrian University of Athens, Attikon University Hospital, 157 72 Athens, Greece; koufonektar@yahoo.com; 6 Department of Obstetrics and Women’s Health, University Medical Center of the Johannes Gutenberg University Mainz, 55131 Mainz, Germany; roxana.schwab@unimedizin-mainz.de; 7 Pathology Unit, Azienda USL-IRCCS di Reggio Emilia, 42123 Reggio Emilia, Italy; magda.zanelli@ausl.re.it; 8 Surgical Oncology Unit, Azienda USL-IRCCS di Reggio Emilia, 42122 Reggio Emilia, Italy; maurizio.zizzo@ausl.re.it; 9 Clinical and Experimental Medicine PhD Program, University of Modena and Reggio Emilia, 41121 Modena, Italy; 10 Department of Medical and Surgical Sciences, Advanced Technologies “G.F. Ingrassia”, 95123 Catania, Italy; giuseppe.broggi@phd.unict.it (G.B.); rosario.caltabiano@unict.it (R.C.); 11 Medical School, National and Kapodistrian University of Atehns, 115 27 Athens, Greece; sophiakalantaridou@gmail.com

**Keywords:** 3D printing, assisted reproductive techniques, ovarian microenvironment

## Abstract

*Background and Objectives:* This article investigates the transformative impact of 3D and bio 3D printing technologies in assisted reproductive technology (ART), offering a comprehensive review of their applications in improving reproductive outcomes. *Materials and Methods:* Following PRISMA guidelines, we conducted a thorough literature search focusing on the intersection of ART and additive manufacturing, resulting in the inclusion of 48 research papers. *Results:* The study highlights bio 3D printing’s potential in revolutionizing female infertility treatments, especially in follicle complex culture and ovary printing. We explore the use of decellularized extracellular matrix (dECM) as bioink, demonstrating its efficacy in replicating the ovarian microenvironment for in vitro maturation of primordial oocytes. Furthermore, advancements in endometrial cavity interventions are discussed, including the application of sustained-release systems for growth factors and stem cell integration for endometrial regeneration, showing promise in addressing conditions like Asherman’s syndrome and thin endometrium. We also examine the role of conventional 3D printing in reproductive medicine, including its use in educational simulators, personalized IVF instruments, and microfluidic platforms, enhancing training and precision in reproductive procedures. *Conclusions:* Our review underscores both 3D printing technologies’ contribution to the dynamic landscape of reproductive medicine. They offer innovative solutions for individualized patient care, augmenting success rates in fertility treatments. This research not only presents current achievements but also anticipates future advancements in these domains, promising to expand the horizons for individuals and families seeking assistance in their reproductive journeys.

## 1. Introduction

The rapid increase in the frequency of assisted reproduction techniques has created a demand for optimizing procedures to ensure successful outcomes. Utilizing 3D printing technologies seems to offer a promising avenue for enhancing and advancing existing techniques, even in everyday clinical practice. Although extensive research exists on the applications of 3D printing in gynecology, there is a noticeable lack of focused literature reviews that address its use in reproductive medicine [1]. However, in the realm of assisted reproduction, a wealth of published data exists for both 3D printing and bio 3D printing. It is important to note that these technologies differ significantly in their scope and the materials they employ. Bio 3D printing utilizes biological materials, sometimes including cellular elements, whereas traditional 3D printing relies on non-biological materials. The selection of these technologies is determined by the particular needs and goals of the reproductive procedures in question. Bio 3D printing holds significant promise in revolutionizing the fields of in vitro fertilization (IVF) and assisted reproductive technology (ART). This cutting-edge technology, which harnesses the precision of 3D printing techniques, offers a range of innovative applications aimed at improving the success rates and outcomes of IVF treatments. These applications include options such as follicle complex culture, printing of ovaries and tissues, printable interventions for the endometrial lining, and even the possibility of printing testicular tissue [2]. On the other hand, conventional 3D printing has valuable applications in the pharmaceutical realm, particularly in the context of personalized treatments. It also serves as a valuable tool for simulating and training fertility-related surgeries and optimizing equipment used in the field of reproductive medicine [3].

The wealth of data available on both technologies and their applications is substantial and growing. However, it is important to note that much of this research is highly specialized and heterogeneous, focusing on specific areas of study within reproductive medicine. Therefore, to gain a better understanding of this information, it is essential to categorize studies based on the technology employed, the desired outcomes, and the specific field they address. This categorization can help researchers and practitioners navigate the diverse and evolving landscape of 3D printing in reproductive medicine more effectively. The purpose of this work is to gather all the data concerning the application of additive manufacturing in assisted reproduction techniques by summarizing both the clinical and laboratory findings as well as the technical characteristics of the printers.

## 2. Materials and Methods

This article adheres to the PRISMA (Preferred Reporting Items for Systematic Reviews and Meta-Analyses) guidelines, and it attempts to provide a thorough and systematic analysis of the matter at hand. Our research methodology involved the following steps:

Literature Search: An extensive search of relevant medical databases for studies from the last decade was performed, with a specific focus on those at the intersection of ART and additive manufacturing. Our search was carried out primarily in three major databases: Cochrane Library, Embase, and PubMed.

Throughout the literature search, the most frequently encountered studies were experimental and clinical research articles focusing on the application of 3D and bio-3D printing technologies in specific areas of reproductive medicine. These included studies on in vitro follicle and oocyte culture, endometrial regeneration, and fertility preservation techniques using bioinks and bioprinting methods. Several studies also examined preclinical applications, utilizing animal models to test the biocompatibility and functionality of 3D-printed constructs in replicating human reproductive environments. Additionally, a significant subset of the literature focused on technical advancements in bioink formulations and bioprinting protocols to optimize cell viability, tissue integration, and structural fidelity. Educational and training simulations leveraging 3D-printed anatomical models for reproductive surgery and ART procedures represented another common study type, reflecting the multidisciplinary nature of 3D printing applications in reproductive medicine.

Keywords: To optimize the search for relevant studies, we utilized an extensive set of keywords, including “3D printing”, “Bio 3D printing”, “additive manufacturing”, “assisted reproduction”, and “IVF”.

Query: The search query utilized across these databases was: ((“Assisted reproductive” AND (“technique” or “techniques”) OR “ART” OR “IVF”) AND (“printing” OR “3D” OR “3D printing” OR “manufacture” OR “manufacturing” OR “bio print” or “3D bio”)).

Inclusion and Exclusion Criteria: To ensure clarity and relevance, the following criteria were applied during the selection process:

Inclusion Criteria: Studies were included if they met the following conditions:

- Directly examined the intersection of additive manufacturing and assisted reproductive techniques.
- Provided clinical or experimental evidence relevant to ART applications, including but not limited to follicle culture, ovary printing, endometrial cavity interventions, and educational or personalized devices.
- Published in peer-reviewed journals within the last ten years.
- Offered quantitative or qualitative outcomes that could inform the potential of additive manufacturing in reproductive medicine.

Exclusion Criteria: Studies were excluded based on the following:

- Focused primarily on additive manufacturing applications outside of ART or reproductive medicine.
- Lacked sufficient detail or context related to ART-specific applications.
- Duplicated findings already represented in other included studies unless additional unique insights were presented.

Snowball Technique and Reference Expansion: Following the initial database search, the snowball technique was employed to identify further relevant papers by examining references within the selected studies. This approach was used to expand our dataset comprehensively, resulting in the inclusion of 48 research papers aligned with the study’s objectives.

**Figure 1:**
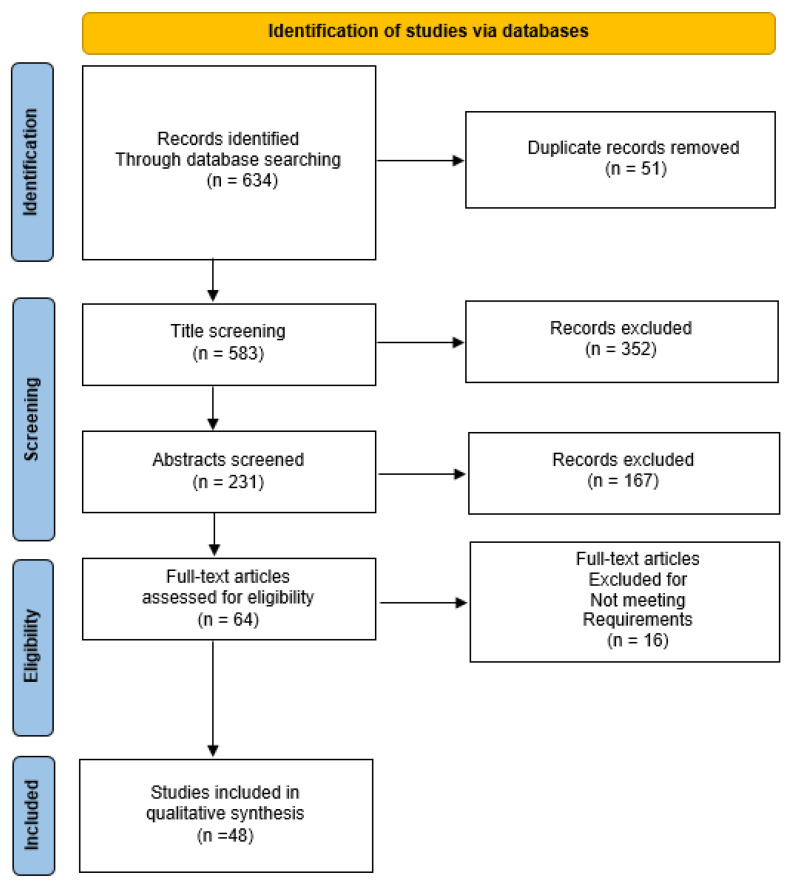
PRISMA diagram indicating the study selection process.

A flowchart detailing the study selection and exclusion process per PRISMA guidelines is presented in Figure 1.

## 3. Results

### 3.1. Bio-3D Printing

#### Follicle Complex Culture and Ovary Printing

To approach the applications of additive manufacturing in human reproduction, it would be logical to start from the cellular level. Undoubtedly, in vitro ovarian follicle culture is a promising addition to assisted reproduction techniques aimed at optimizing the methods we have for in vitro culture, growth, and maturation of primordial oocytes and their subsequent utilization for reproductive purposes [4,5]. It is known from human physiology that the ovarian cortex is colonized by immature oocytes in meiotic arrest, which are called primordial oocytes [6]. In vivo, either in the context of the menstrual cycle or after the application of some stimulation protocol in the context of ART, the hormonal stimuli exerted on the ovary lead to oocyte maturation. In the normal menstrual cycle, one follicle becomes Graafian, while on the contrary, in stimulation protocols, multiple mature follicles are retrieved [7]. Another alternative, which was developed mainly for reasons of urgent fertility preservation in young cancer patients, is ovarian cortex extraction and then maturation of the follicles in vitro [8,9].

The ovarian tissue is either directly subjected to follicle extraction and maturation or preserved through cryopreservation until it can be used at the patient’s request. It is extremely important to understand that each in vitro maturation method approaches the whole follicle/oocyte complex, as it is well established that oocyte growth and meiotic competence depend on the gap junctions between the oocyte and granulosa cells [10]. Preserving the complex 3D architecture and the granulosa–oocyte interaction may be vital for successful in vitro follicle maturation, ensuring the gap junctions remain functional. [11].

Initially, 2D culture systems were primarily used for research purposes but demonstrated limited success with human follicles. In traditional two-dimensional (2D) tissue culture systems, follicles often lose their structure, with granulosa cells—which support the growing oocyte—migrating away, leaving the oocyte exposed and unable to complete maturation [12]. This issue is particularly pronounced with human primordial follicles, which may require up to three months in culture [13]. As a result, the need for a 3D culture system became apparent to maintain the spherical morphology of the ovarian follicle and preserve essential cell–cell and cell–matrix interactions within the surrounding stromal tissue, thus enabling successful follicular maturation.

In response to this need, bio 3D printing technology has made significant contributions. This technique allows for the precise fabrication of biological 3D structures that contain cells, extracellular matrix scaffolds, and biochemical factors [14], better replicating the human in vivo microenvironment compared to 2D systems. However, some reports suggest that cells may experience damage during the printing process due to shear stress [15]. To address this, selecting appropriate bioinks and bioprinters is crucial to supporting cell viability. Commonly used bioinks include naturally derived biomaterials like polysaccharides, fibrin, and collagen, all of which have excellent biocompatibility. Notably, studies have identified the decellularized extracellular matrix (dECM) as the optimal matrix for artificial ovaries [16], as synthetic polymers lack the full range of properties found in dECM [17,18]. Both from the application of dECM [19,20] and hydrogel [21] in animal experiments, very interesting feedback with successful in vitro follicular culture was obtained. Of great interest is the work of Hassanpour et al. in 2015, who, in addition to animal follicles, tried the 3D culture of human follicles in dECM [22]. The survival and development of human pre-antral follicles within a decellularized ovarian scaffold have demonstrated the potential of the scaffold to support follicle survival and growth, a very promising factor in in vitro maturation applications [23,24].

The ultimate clinical application of the above is the printing of functional ovaries. First, Laronda et al. in 2017 reported the printing of functional ovaries in mice using Decm as a bioink [19]. Two more 3D printing efforts have since been announced. The first uses an alternative bionk option that combines gelatin methacryloyl (GelMA), alginate, and GelMA-alginate bioinks (Wu et al., 2022) [25]. The second refers to a 3D-bioprinted engineering ovary composed of ADSCs (adipose-derived stem cells) that construct an early vascular microenvironment, which in combination with a drug-free IVA enhances follicle growth and maturation of impaired ovarian function in the POI rats [26].

### 3.2. Endometrial Cavity Interventions

Achieving pregnancy through the application of ART methods is the result of many parameters [27]. Optimization of preimplantation screening methods has greatly reduced implantation failure due to fetal factors [28]. On the other hand, the complexity of the factor of endometrial receptivity has been highlighted. There is a limited number of studies that focus exclusively on the effect of endometrial treatment on the achievement of embryo implantation and clinical pregnancy, and there is a shortage of randomized controlled trials and evidence-based studies with large sample sizes [29]. However, the data to date show higher rates of pregnancy achievement in cases of healthy endometrium without fibrosis and infection [30]. The main pathological entities that cause inflammation, fibrosis, and endometrial adhesions are Asherman’s syndrome and endometritis [31,32]. On the other hand, endometrial thickness plays a crucial role in successful implantation [33]. Despite various treatments suggested in the literature, such as transvaginal sildenafil citrate, pentoxifylline combined with tocopherol, and high-dose estrogen, many patients still fail to achieve the desired endometrial thickness of over 7 mm. This often leads to either the cancelation of embryo transfer with subsequent cryopreservation or proceeding with the transfer despite a thin endometrium, thereby accepting reduced pregnancy success rates [34].

It is obvious that maintaining the proper endometrial characteristics is crucial in the attempt to conceive through ART, and this is the reason why extensive study has been conducted in this area. In this direction, research has been conducted to pinpoint the precise function of cytokines and endometrial growth factors [35]. In particular, granulocyte colony-stimulating factor (G-CSF), a member of the cytokine family that includes other colony-stimulating factors, has garnered significant attention [36]. It has been shown to influence embryo implantation, embryonic growth, stromal cell decidualization, and the invasion of trophoblast cells into maternal tissue [37]. Research has demonstrated that G-CSF perfusion therapy is beneficial for patients experiencing recurrent implantation failure, suggesting the positive effects of G-CSF on the endometrium. However, G-CSF has a relatively short half-life in the human body [38], and its concentration at the site of injury is often low due to internal migration. Consequently, further research into sustained-release drug delivery systems with high local concentrations is warranted. Wen et al. (2022) developed a 3D-printed scaffold using gelatin and sodium alginate, incorporating a G-CSF sustained-release microsphere (SRM) system with an average diameter of 9.68 m [39]. The specific substance was administered to rats with endometrial adhesions, resulting in local regeneration of the endometrium and maintenance of the injured endometrium’s pregnancy-related activities [39].

The infusion of stem cells into the endometrium adds another dimension to endometrial regeneration. Stem cells derived from menstrual blood (MenSCs), umbilical cord mesenchymal stromal cells (UC-MSCs), bone marrow (BMSCs), embryonic stem cells (ESCs), and adipose tissue (ADSCs) have all been utilized [40] in order to optimize endometrial cell growth. A 3D bioprinter was employed to create a hydrogel scaffold loaded with human-induced pluripotent stem cell-derived mesenchymal stem cells (hiMSCs), which was subsequently implanted in vivo [41]. The team discovered that it might stimulate endometrial tissue repair as well as endometrial cell regeneration, including mesenchymal, epithelial, and nerve cells [41]. Additionally, Feng et al. developed composite tissue hydrogels with varying ratios of gelatin methacrylate (GelMA) and collagen methacrylate (ColMA) using 3D bioprinting technology [42]. In vitro experiments demonstrated that stem cells could be continuously released for at least 7 days during the 3D printing process utilizing this hydrogel. In vivo tests revealed that a GelMA/ColMA/hAMSC (human amniotic mesenchymal stem cells) hydrogel model was effective in preventing intrauterine adhesions in a rat IUA model [42].

### 3.3. Genital Malformations

Treatment of genital abnormalities is important to ensure female fertility and the success of IVF techniques. The technology of bio 3D printing has primarily been used in cases of vaginal tissue loss, focusing on vaginal reconstruction using non-vaginal tissue sources. Hou et al. [43] developed a bioink with excellent biocompatibility by combining gelatin and sodium alginate with decellularized animal vaginal epithelial tissue. This bioink was used to print 3D scaffolds for the culture of bone marrow mesenchymal stem cells (BMSCs). In vitro experiments showed that BMSCs had a high survival rate (95%) in the 3D scaffolds on the first day, maintaining strong viability after a week. In vivo, vaginal epithelial cells were successfully cultured in mice, suggesting that 3D printing technology could offer a promising new approach for developing vaginal tissue replacements.

### 3.4. Male Factor Infertility

Research on 3D bioprinting for the testis is still in the stage of development. Autologous or allogeneic transplantation is a treatment option for individuals with severe bilateral testicular atrophy, small bilateral testes, or testicular agenesis. Since prepubertal males do not produce sperm, cryopreserved immature testicular tissue (ITT) transplantation may offer a promising solution to restore fertility in young males. However, studies have shown that chemotherapy and radiotherapy used in childhood cancer treatments can irreversibly impact fertility in adulthood. Regardless of the cryopreservation method, the amount of spermatogonial cells significantly decreased after ITT transplantation [44]. To address this issue, Poels et al. embedded ITT in hydrogels containing VEGF nanoparticles and evaluated the seminiferous tubule integrity, hematopoietic reconstitution, and spermatogonia recovery using immunohistochemistry [45]. Their results demonstrated that alginate hydrogels with nanoparticle growth factors significantly improved spermatogonia recovery and could potentially simplify tissue transplantation during cryopreservation.

#### 3.4.1. Testicular Tissue Engineering

One of the foremost areas of research in male infertility is the bioprinting of testicular tissue. Severe cases of infertility, such as testicular atrophy or agenesis, present complex treatment challenges. Recent studies have explored the use of 3D-bioprinted scaffolds to support testicular tissue regeneration, particularly for prepubertal boys undergoing cancer treatments that compromise fertility [46]. Poels et al. demonstrated that embedding cryopreserved immature testicular tissue (ITT) in alginate hydrogels containing vascular endothelial growth factor (VEGF) nanoparticles significantly improved spermatogonial recovery post-transplantation, promoting seminiferous tubule integrity and reconstitution of hematopoietic cells [44].

#### 3.4.2. Sperm Production and Maturation Support

3D printing technology has enabled the creation of microfluidic devices that simulate the seminiferous tubule environment, supporting the culture and maturation of spermatogonial stem cells (SSCs). These devices aim to mimic the natural gradient of nutrients, oxygen, and hormonal cues found in the testis, facilitating in vitro spermatogenesis. Research has shown that such devices can help mature SSCs into functional sperm-like cells, offering potential fertility solutions for men with azoospermia or those who have undergone sterilizing treatments [47].

#### 3.4.3. Sperm Selection and Sorting Devices

Another notable application of 3D printing in ART for male infertility is in sperm selection. Traditional sperm selection methods, such as swim-up and density gradient centrifugation, have limitations in isolating high-quality sperm with intact DNA. Advances in microfluidic sperm selection devices—such as the biomimetic sperm selection platform (MSSP) developed by Vasilescu et al.—allow for enhanced sperm quality by selecting for motility, morphology, and reduced DNA fragmentation. These 3D-printed devices simulate the natural barriers sperm must navigate, selecting the most viable cells and yielding improved outcomes in ART procedures such as in vitro fertilization (IVF) and intracytoplasmic sperm injection (ICSI) [48].

#### 3.4.4. Cryopreservation and Storage Solutions

Additive manufacturing has also contributed to improved cryopreservation techniques for sperm. Innovative 3D-printed cryopreservation devices are being designed to minimize cellular damage and enhance post-thaw viability, which is critical for long-term sperm storage. These devices use compartmentalized structures to evenly distribute cryoprotectants, thereby reducing osmotic shock and enhancing sperm recovery rates. For men requiring fertility preservation, particularly those undergoing treatments that impact reproductive health, such devices offer a more effective approach to maintaining sperm integrity over time [49].

#### 3.4.5. Future Directions in Male Reproductive Bioprinting

The potential of bioprinting in male reproductive health extends beyond these initial applications. Future research may focus on engineering complete testicular structures capable of producing functional sperm, supported by advances in stem cell biology and microenvironment engineering. As bioprinting technology evolves, it may also enable the creation of personalized devices and systems for male fertility treatments, addressing the unique physiological and genetic factors that contribute to infertility in men.

## 4. Conservative 3D Printing

### 4.1. Endometriosis Treatment

Endometriosis, i.e., the presence of endometrial tissue in locations other than the endometrial cavity, is mainly related to pain and alteration of the woman’s quality of life. In addition to chronic pain, several studies associate endometriosis with female infertility and an increased likelihood of failure to achieve pregnancy through ART techniques. So far, the gold standard of treatment is surgical treatment; however, there are also many pharmaceutical options, most of which are related to the suppression hypothalamic–pituitary–ovaries axis and the corresponding estrogenopenia, with the result that they are not compatible options with the preservation of women’s reproductive capacity.

An alternative pharmaceutical approach involves the use of drugs that modulate TGF-β, such as pirfenidone. Pirfenidone is a pyridine-based medication that can be used for the treatment of pulmonary fibrosis. In 2023, Teworte et al. published an extremely interesting approach in which risperidone was administered to patients in the form of 3D-printed mucoadhesive vaginal ovules [50]. In further detail, a semisoft alginate-based vaginal suppository, created using semisolid extrusion additive manufacturing, has shown greater effectiveness compared to traditional vaginal ovules made with standard excipients. The 3D-printed ovule demonstrated a controlled release of pirfenidone during both standard and biorelevant in vitro release tests and also exhibited superior mucoadhesive properties in ex vivo evaluations. In order to reduce the metabolic activity of the endometriotic epithelial cell line (12Z), a continuous 24 h exposure to pirfenidone was required, underscoring the importance of a sustained-release formulation for this drug.

Another application of 3D printing for the treatment of endometriosis is that of adjunctive preoperative printing of models of the patient’s pelvis for careful preoperative planning and localization of endometriosis foci. Specifically, in 2017, Ajao M. et al. described a 3D-printed model of a deep infiltrating endometriosis (DIE) nodule following its laparoscopic removal [51]. Then, followed an attempt to model the female pelvis with a lot of work related to pelvic 3D printing, which contributed to the optimization of the casts and the development of related image printing technology based on MRI and ultrasound imaging technology [52,53]. In 2022, the first case series was announced, describing the preoperative modeling of the pelvis of 7 women with DIE, with encouraging results regarding the postoperative course and remission of the patients’ chronic pain symptoms compared to the control group [54]. The above cases do not include studies aimed at reproduction, but in any case, the treatment of endometriosis is an approach that must be considered individually in each case of infertility.

### 4.2. Three-Dimensional Printed Simulators for Educational Purposes and Personalized Services

Like in many medical specialties, reproductive doctors require not only a strong theoretical foundation and daily clinical practice but also advanced technical skills. With the increasing complexity of clinical cases and the growing body of knowledge to master, simulation-based learning has become essential. Repeated practice often enhances both performance and confidence [55]. Three-dimensional printing plays a pivotal role in the development and design of digital simulators. Ceccaldi et al. created 3D-printed uterine models with modified cavities, which can be used for practicing embryo transfer and virtual hysteroscopy, along with addressing the challenges they present [56]. This specific application can, on the one hand, contribute to the training of the medical staff but also to the understanding of the patient’s anatomy in order to utilize all the possibilities so that the embryo transfer attempt does not fail. Another 2 clinical applications of the educational models refer to laparoscopic myomectomy and genital malformation correction, which potentially find application in the reproduction field [57,58].

### 4.3. Merging 3D-Printed IVF Technology

Three-dimensional printing technology is becoming increasingly prominent in the technical aspects of Assisted Reproductive Technology (ART) and is gradually replacing traditional methods. A notable example is the introduction of the nano-liter perfusion microfluidic device, manufactured entirely through two-photon polymerization (2PP), into daily clinical practice for dynamic cell culture. Microfluidic devices have been developed over the years to enable dynamic fluid delivery during in vitro cell culture, with the added benefit of allowing cell retrieval by removing parts of the device. These devices are designed to handle and analyze small fluid volumes at the microscale, making them particularly useful for sperm sorting and preparation, oocyte manipulation, embryo culture and assessment, hatching, cryopreservation, and preimplantation genetic testing [59].

Three-dimensional printing has enabled the creation of a two-part microfluidic device capable of managing nanoliter volume flow rates, entirely manufactured using 2PP. This printing method has allowed for the production of complex geometries previously unattainable in biologically compatible microfluidic cell culture devices. The nest-and-cradle design facilitates the delivery of culture media, dyes, and biological molecules, promoting quantifiable, physiologically relevant changes in cell growth and morphology while also allowing for cell retrieval for further functional analysis. Ongoing advancements aim to enhance this technology for automated in vitro embryo culture and three-dimensional cell culture at the microscale [60].

Additionally, in the realm of embryo culture, a new 3D-printed device has been developed that combines minimum volume cooling vitrification with the capacity to vitrify a large number of embryos simultaneously, marking a significant step forward in cryopreservation technology [48].

Another remarkable application of 3D printing in ART is the creation of personalized IVF instruments. A prime example is a 3D-printed sperm selection device that features a network of 560 microchannels. This device selects high-quality sperm with significantly improved DNA integrity and morphology through a single-step process. The design leverages the increased contact area between the semen sample and a fresh buffer, facilitating a high-throughput selection mechanism [61].

Additionally, Vasilescu et al. recently introduced a biologically inspired microfluidic sperm selection device (MSSP), which simulates the sperm’s natural journey toward selection using multiple criteria [62]. Sperm are initially selected based on their motility and boundary-following behavior, followed by screening for apoptotic markers. This method yields over 68% more motile sperm compared to previously reported techniques, with a lower incidence of DNA fragmentation and apoptosis. Furthermore, sperm processed through the MSSP demonstrated higher motility recovery post-cryopreservation than those obtained through SU or neat semen methods.

## 5. Technical Review

**Figure 2:**
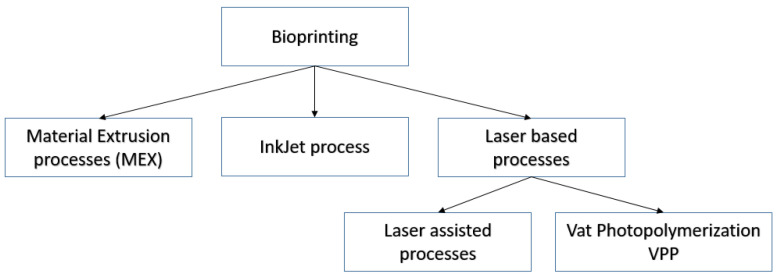
Summarizing 3D printing technical information.

Without a doubt, bioprinting represents a groundbreaking tissue engineering (TE) approach with immense potential as a platform for constructing and fabricating in vitro tissues. Almost all the current bioprinters make use of traditional, well-established additive manufacturing (AM) technologies like material extrusion (MEX) and vat photopolymerization (VPP) [63]. Regardless of the printing process, all printers use bioinks with different, process-specific material properties such as viscosity, density, and adhesion requirements. Moreover, process parameters like printing speed, temperature, material deposition rates, and laser power also need to be adjusted depending on machine type and component fabrication (Figure 2).

Bioprinting’s increasing applications have contributed to the development of a variety of bioprinting technologies and companies, from the Industry Pioneer Organovo to companies and scientific teams focusing on different aspects such as hardware, bioink composition, or end-use applications. Bioprinters can be divided into three categories based on size and cost. Small, portable bioprinters for entry-level applications like INKREDIBLE + of CELLINK and 3Dynamic Alpha of 3Dyanmic Systems. Medium-sized bioprinters for larger and more complex applications like NovoGen MMX Bioprinter™ of Organovo and RX1™ BIOPRINTER of Aspect Biosystems. Last but not least, large-scale bioprinters with the highest cost for high complexity and volume applications like NGB 17.03 of Poietis and ALPHA-CPT1 of SunP Biotech International.

Besides the growing number of companies and institutions investing in research and development for bioprint technology, both hardware development and bioink composition, still a lot of research is needed, especially in the optimization of process parameters and material properties for bioprinters and bioinks. Design for additive manufacturing (DfAM) is a methodology, widely used for industrial additive manufacturing, aiming to understand and overcome AM limitations while utilizing AM advantages. Similarly, biomedical researchers are aiming to better understand the effect of design, printer, and bioink properties on bioprintability, mechanical strength, and structural integrity, as well as the degradation behavior of scaffolds, artificial tissue, and bioprosthetics. Wu, T. et al. evaluated the use of three different bioinks (GelMA, Alginite, and GelMAAlginite) for scaffold fabrication using a SUNP BIOMAKE 2 bioprinter and cell-laden 3D printing, which were utilized to create artificial ovaries using commercial ovarian tumor cell lines and ovarian somatic cells. Alginite and GelMA alginate bioinks were eliminated after evaluation, whereas GelMA-printed scaffolds revealed promising results [63]. On the other hand, in cell-laden 3D printing, extensive primary cell death was observed within the grids, and therefore more research is needed. Reid, J.A. et al., besides introducing a process of converting an inexpensive 3D printer to a bioprinter, giving the opportunity to researchers to obtain a low-cost, reliable, and open-source bioprinter, focused their research on optimizing micro-needle geometry and bioink rheological properties in order to minimize needle clogging along with cell damage due to mechanically induced stress and strain phenomena [14]. Their research revealed that altering the design of the needle geometry from a ‘luer-lock’ type to a triangular (conical) configuration leads to a non-Newtonian, constant volumetric flow that minimizes the applied forces and cell damage.

## 6. Limitations and Future Directions

While this review demonstrates the promising advancements in additive manufacturing applications within assisted reproductive technology (ART), several limitations within the current body of research should be acknowledged.

### 6.1. Limitations of Current Research

- Lack of Standardization: Studies often vary widely in methodology, particularly regarding bioink formulations, printing parameters, and evaluation criteria. This lack of standardization makes it challenging to directly compare outcomes across studies and may hinder the translation of experimental findings to clinical practice.
- Limited Clinical Data: Although preclinical studies using animal models and in vitro experiments show potential, there is a scarcity of robust clinical trials examining the safety and efficacy of 3D-printed constructs in human reproductive applications. This limits the immediate clinical applicability of findings and suggests a need for more extensive human-based studies.
- Bioink and Bioprinter Constraints: Current bioinks and bioprinter technologies, while innovative, still face challenges in fully replicating the complex microenvironment of human reproductive tissues. Issues such as cell viability during printing, structural integrity of printed constructs, and long-term biocompatibility remain areas where significant improvements are needed.
- Ethical and Regulatory Barriers: The use of bio 3D printing, particularly in reproductive medicine, introduces ethical considerations, especially when manipulating human tissues or cells. Furthermore, regulatory guidelines are still evolving, and the approval process for bioprinted materials and devices remains complex.

### 6.2. Future Directions

- Development of Optimized Bioinks: Future research should prioritize developing bioinks that better mimic the extracellular matrix of reproductive tissues, enhancing cell viability, functionality, and tissue integration. Innovations in dECM-based and multi-material bioinks are particularly promising avenues.
- Longitudinal Clinical Trials: Conducting longitudinal studies with larger patient cohorts will be essential to validate the safety, effectiveness, and reproducibility of bioprinting applications in ART. These studies can provide data on long-term outcomes and potential side effects, thereby facilitating clinical translation.
- Enhanced Bioprinter Precision and Scalability: Ongoing advancements in bioprinter technology, such as multi-nozzle systems and finer resolution capabilities, will allow for more precise fabrication of complex tissues. Additionally, developing scalable processes that can transition from lab to clinic is necessary for widespread clinical adoption.
- Exploration of Stem Cell Integration: The incorporation of stem cells within bio-printed constructs for endometrial and ovarian regeneration presents a promising future direction. Research focusing on the differentiation and survival of stem cells within printed tissues could pave the way for new treatments for infertility.
- Ethical and Regulatory Frameworks: Addressing ethical concerns and establishing clear regulatory pathways for bio 3D printing in reproductive medicine is critical. Future studies should engage with bioethics experts and policymakers to foster a responsible and legally compliant framework for these technologies.

## 7. Discussion

The amalgamation of 3D printing technology within the domain of reproductive medicine stands as a vanguard with immense potential, already marking significant progress across various facets of assisted reproduction. Particularly, bio 3D printing has emerged as a pioneering instrument with transformative capacities. The realms of follicle complex culture and ovary printing have introduced a revolutionary paradigm in addressing female infertility. These technologies hold the potential to refine the in vitro culture and maturation of primordial oocytes, a pivotal juncture in assisted reproduction.

The development of 3D-printed ovarian follicle complexes, which replicate the natural ovarian microenvironment, represents a major breakthrough for in vitro oocyte maturation. Unlike traditional 2D culture systems, 3D printing preserves critical cell–cell and cell–matrix interactions, resulting in improved oocyte maturation and fertility outcomes. This technology offers promising solutions for patients with diminished ovarian reserves or those requiring fertility preservation. Moreover, when it comes to endometrial regeneration and receptivity, restoring healthy endometrial tissue is critical for successful ART outcomes, especially in cases of Asherman’s syndrome and thin endometrium. Bioengineered scaffolds, growth factor delivery systems, and stem cell therapies have demonstrated effectiveness in promoting endometrial regeneration and reducing adhesions, thereby enhancing embryo implantation success rates and reducing implantation failures.

The utilization of decellularized extracellular matrix (dECM) as a bioink has yielded promising outcomes in the construction of artificial ovaries that faithfully replicate the natural microenvironment. Endometrial cavity interventions, orchestrated through 3D bioprinting, present innovative avenues for augmenting endometrial receptivity and com-batting conditions like Asherman’s syndrome and thin endometrium. The emergence of sustained-release systems for growth factors like granulocyte colony-stimulating factor (G-CSF), along with the incorporation of stem cells for endometrial regeneration, marks an exciting advancement in the field. The purview of genital malformations, specifically vaginal reconstruction, has derived substantial benefits from bio 3D printing, offering renewed hope to women grappling with such challenges.

While research has predominantly concentrated on female infertility, emerging work on male factor infertility through 3D bioprinting offers promising avenues for restoring fertility, particularly in cases such as testicular agenesis and atrophy. Complementing these advancements, conventional 3D printing contributes significantly by creating educational models, personalized tools, and microfluidic platforms tailored to in vitro fertilization (IVF) processes. This technology supports the training of healthcare providers, refinement of surgical techniques, and enhancement of precision across reproductive procedures. Innovations such as microfluidic devices for sperm selection and bioengineered testicular tissue hold the potential to improve sperm quality in ART, boosting fertilization rates and minimizing genetic abnormalities.

Also, the advancement of these technologies brings educational and training enhancements to the existing processes. Three-dimensional-printed anatomical models and simulation tools provide essential training for reproductive specialists, improving practitioner proficiency in complex procedures such as embryo transfers and fertility surgeries. This leads to higher success rates and better patient outcomes. Patient-specific models also foster deeper patient understanding and informed decision-making.

Nevertheless, the application of bio 3D printing in reproductive medicine raises important ethical and regulatory issues, particularly around tissue manipulation and the safety of bio-printed constructs. Establishing comprehensive regulatory frameworks and prioritizing ethical considerations will be critical to ensuring safe and effective clinical applications.

## 8. Conclusions

Both bio-3D printing and conventional 3D printing collectively contribute to the dynamic landscape of reproductive medicine. They herald opportunities for individualized care, augmented success rates, and pioneering strategies to grapple with intricate fertility challenges. The ongoing research and development in these domains persistently challenge the boundaries of possibility, promising a brighter horizon for couples seeking assistance in their reproductive journeys. As these technologies mature, one can anticipate even more profound ramifications for the field, ultimately disseminating hope and joy to individuals and families grappling with infertility.
